# Genetic Diversity, Admixture and Analysis of Homozygous-by-Descent (HBD) Segments of Russian Wild Boar

**DOI:** 10.3390/biology11020203

**Published:** 2022-01-27

**Authors:** Olga Kostyunina, Aleksei Traspov, Alexander Economov, Ivan Seryodkin, Aleksandr Senchik, Neckruz Bakoev, Yuri Prytkov, Nikolay Bardukov, Igor Domsky, Tatiana Karpushkina

**Affiliations:** 1 Federal Research Center for Animal Husbandry Named after Academy Member LK. Ernst, 142132 Dubrovitsy, Russia; a.traspov@sistemabiotech.ru (A.T.); nekruz82@bk.ru (N.B.); prytkov_y@mail.ru (Y.P.); bardukv-nikolajj@mail.ru (N.B.); t.kriz@ya.ru (T.K.); 2 Sistema-Biotech, Limited Liability Company, 109235 Moscow, Russia; 3 Professor Zhitkov Federal State Budgetary Russian Research Institute of Game Management and Fur Farming, 610000 Kirov, Russia; aconom86@mail.ru (A.E.); igordomsky@mail.ru (I.D.); 4 Pacific Geographical Institute, Far Eastern Branch of the Russian Academy of Sciences, 690041 Vladivostok, Russia; seryodkinivan@inbox.ru; 5 Autonomous Noncommercial Organization of Higher Education, Moscow International University, 125040 Moscow, Russia; senchik_a@mail.ru

**Keywords:** wild boar, admixture, domestic pig, genetic diversity, Homozygous-by-Descent

## Abstract

**Simple Summary:**

The wild boar is one of the most common wild animals. On the territory of Russia, there are two subspecies of the wild boar—European and Asian. At the beginning of the 20th century, the wild boar in the European part of Russia was practically exterminated. Later the population was restored by importing animals from other regions and by self-repopulation. The aim of our research was to assess the population structure of the Russian wild boar in comparison with the wild boar from other regions of the world and to determine the level of autozygosity, which allows us to determine the state of the population. We found traces of introgression of the Asian wild boar into the European one due to, migration of the wild boar in the population recovery process. Further analysis for genetic influx into Russian wild boar population identified four samples in which more than 10% of the genome belonged to domestic pigs. The Homozygous-by-Descent (HBD) Segments evaluation showed a low level of autozygosity in comparison with the aggregate sample of the European wild boar. Based on our genetic evaluation, we concluded that the population of the Russian wild boar of the European and Asian subspecies are characterized by a sufficient level of genetic diversity.

**Abstract:**

The wild boar is the wild ancestor of the domestic pig and one of the most common species of ungulates. At the beginning of the 20th century, the wild boar was practically exterminated in the European part of Russia. In the period 1935–1988, 7705 boars were caught in various regions of the European part of Russia, the Far East, Ukraine, Belarus, Kyrgyzstan, Kazakhstan, Latvia, Lithuania, Estonia, Tajikistan and resettled in the territory of Russia. Asian and European wild boars dwell the territory of Russia. The aim of our research was to study the genetic diversity and structure of wild boar populations in different regions of Russia using genome-wide genotyping. We have determined the genetic distances, population structure, parameters of genetic diversity and significantly expanded our understanding of the genetic state of the Russian wild boar. For the first time, we calculated autozygosity of the wild boar of the European and Asian subspecies using Homozygous-by-Descent (HBD) Segments analysis, which is important in terms of population recovery. We also found evidence of hybridization between Russian wild boar and domestic pigs. A group of European wild boars showed introgression of the Asian boar into population. The mean level of the inbreeding coefficient in European wild boar was higher than in Asian wild boar, and combined groups of the European boar had higher inbreeding coefficient than Russian wild boars. These results obtained can be used in population management.

## 1. Introduction

The wild boar (*Sus scrofa*, Linnaeus, 1758, synonyms *Sus attila*, *Sus lybicus, Sus algira*) is the wild ancestor of the domestic pig and one of the most common species of ungulates. It inhabits most of Europe, the entire Middle East up to the Zagros Ridge and North Africa [1]. This species has a native distribution ranging from the tropical region of Asia to Siberian forests, including semi-desertic and temperate ecosystems. Such expanded ecological presence is probably linked to the particular life-history traits of the species, which is characterized by high population turnover, a peculiar pattern of elasticity of demographic parameters [2], and survival resilience [3]. The ancestral forms of the European and Asian subspecies are estimated to have diverged from each other between 500,000 and one million years ago [4,5].

At the beginning of the 20th century, the population of wild boar severely decreased in the European part of Russia [6]. The resettlement of wild boars in the western part of the European territory of Russia began in the 1940s. In the 50s and 60s the wild boar settled in the northwestern and central regions of the country, in the 70s it expanded to the northern and northeastern regions (Arkhangelsk, Vologda, Kostroma, Kirov, Perm regions, Udmurtia) [7]. In the period 1935–1988, 7705 boars were caught in various regions of the European part of Russia, the Far East, Ukraine, Belarus, Kyrgyzstan, Kazakhstan, Latvia, Lithuania, Estonia, Tajikistan and and resettled in the territory of Russia. The wild boar is an aboriginal species of the Russia`s fauna, which has mainly naturally restored its state in the 20th century [6]. In Russia, the wild boar is distributed in the European part (up the north of Karelia and middle Urals), in the North Caucasus, the Caspian region, the west and south of Western Siberia, the south of Central Siberia, in the Cisbaikalia, Transbaikalia, the Amur region, Primorsky Krai.

During the period from 2016 to 2019, the number of wild boars decreased from 338,900 to 286,400. The change in the number and density of the wild boar population was due to intensive measures to regulate their number in the regions of the Central, Southern, North Caucasus and Volga Federal Districts in order to prevent the emergence and spread of the African swine fever epizootic (ASF) [8].

Currently, due to the spread of ASF, there is a threat of reduction in the number of the wild boar. Wild boar plays an essential sanitary, protective and reforestation role. Its depopulation can result in harmful ramifications in forest ecosystems [9].

The use of molecular genetic methods makes it possible to characterize animal populations and determine the degree of their genetic differentiation. Various types of DNA markers are used to characterize the wild boar populations from Russia: mitochondrial DNA [10,11,12], Y-chromosome [12,13], STR [14,15,16,17], SNP [18,19,20].

The wild boar population of the European part underwent significant changes in size. Despite a number of studies aimed at characterization of the modern population of the Russian wild boar, its origin and genetic structure of the Russian population have not been sufficiently studied. Since the level of autozygosity has not been fully removed from population, the use of Genome-wide methods allow the study of the structure of population, possible introgression of domestic pigs into wild pigs and determine the inbreeding coefficient.

Studying of spreading HBD and non-HBD segments into genome has become very popular in recent times due to its wide range of applications [21]. HBD segments can help to estimate the level of inbreeding, to study inbreeding depression, identify recessive deleterious variants and to determine population structure [22,23,24].

Assessment of genetic diversity and population structure is a fundamental task. This is important for understanding the evolutionary history of its origin, and to provide important information for the conservation and management of biodiversity [25,26]. In this aspect, the assessment of the wild boar is of particular interest, since both of its subspecies—Asian and European wild boars live on the territory of Russia. However, the origin and genetic structure of the Russian population has not been sufficiently studied. In this regard, the aim of our research was to study the genetic diversity and structure of wild boar populations in different regions of Russia using genome-wide genotyping.

## 2. Materials and Methods

### 2.1. Animals

**Figure 1:**
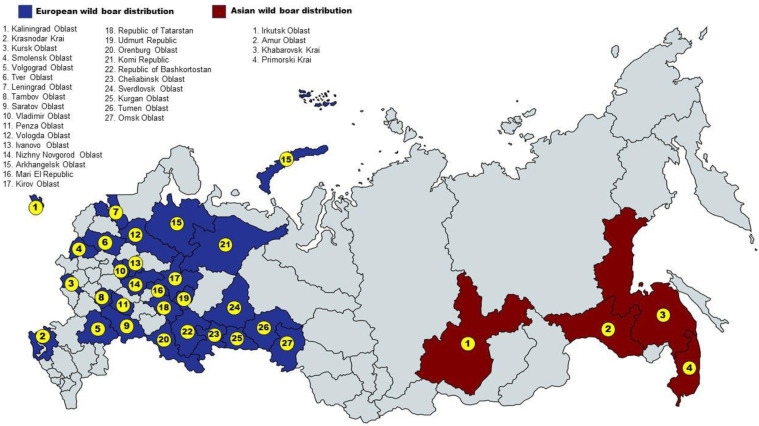
Map with sampling locations.

No animal has been sacrificed for the purposes of this study. Wild boar samples came from regular hunting, according to the Russian (national and regional) laws. In total, 166 samples of wild boar (*S. scrofa*) were taken from 31 regions, which, depending on the territorial location, were assigned to European or Asian part of Russia and one region of Ukraine (Figure 1): RUEU (Kalinigrad (*n* = 1), Kharkov (*n* = 2), Volgograd (*n* = 8), Krasnodar (*n* = 5), Nizhny_Novgorod (*n* = 5), Mari_El (*n* = 1), Tambov (*n* = 1), Penza (*n* = 3), Kursk (*n* = 2), Leningrad (*n* = 6), Ivanovo (*n* = 1), Tver (*n* = 1), Saratov (*n* = 1), Vladimir (*n* = 4), Smolensk (*n* = 4), Bashkortostan (*n* = 2), Chelyabinsk (*n* = 2), Kirov (*n* = 5), Kurgan (*n* = 7), Omsk (*n* = 3), Orenburg (*n* = 2), Sverdlovsk (*n* = 3), Tatarstan (*n* = 2), Udmurt (*n* = 4), Vologda (*n* = 4), Arkhangelsk (*n* = 9), Komi (*n* = 1), Tyumen (*n* = 10)) and RUAS (Primorsky (*n* = 43), Amur (*n* = 14), Khabarovsk (*n* = 8), Irkutsk (*n* = 2)).

Genomic DNA was extracted from the tissue of each animal using NEXTTEC kits (Nexttec Biotechnologie GmbH, Hilgertshausen, Germany) and “DNA-extran-2” (OOO ‘Syntol’, Moscow, Russia) according to the manufacturer’s protocols. The quantity and quality of DNA were assessed using a Qubit 2.0 fluorometer (Invitrogen/Life Technologies, Waltham, MA, USA) and a NanoDrop8000 spectrophotometer (ThermoFisher Scientific, Waltham, MA, USA). Then DNA samples were diluted to a concentration of 50 ng/mL for genotyping.

### 2.2. Data Processing and Data Analyses

The samples were genotyped with the Illumina PorcineSNP60 BeadChip (*n* = 116) and GeneSeek^®^ GGP PorcineHD BeadChip (*n* = 50) on an iScan System (Illumina Inc, USA) following the manufacturer’s protocol, respectively. Additionally, we collected genotyping data from 706 samples, including 422 wild boars from Tunisia (TUN, *n* = 7), Belgium (BELG, *n* = 4), Luxemburg (LUX, *n* = 4), Portugal (POR, *n* = 11), Spain (SPA, *n* = 7), France (FRA, *n* = 28), Germany (GER, *n* = 60), Netherlands (NETH, *n* = 62), Greece (GRE, *n* = 5), Italy (ITA, *n* = 15), Sardinia Islands (SAR, *n* = 99), South Balkan wild boars (SBWB, *n* = 20), Sweden (SWE, *n* = 2), Finland (FIN, *n* = 5), Serbia (SER, *n* = 4), Bulgaria (BUL, *n* = 5), Croatia (CRO, *n* = 16), Slovenia (SLO, *n* = 20), Poland (POL, *n* = 14), Thailand (THAI, *n* = 5), Chinese wild boar (CNWB, *n* = 29), 281 Domestic pigs of Duroc (DUR, *n* = 79), Landrace (LDR, *n* = 129), Large White (LWT, *n* = 76) breeds from the Dryad Digital Repository [27,28,29,30], which were genotyped on the Illumina PorcineSNP60 BeadChip panel.

In order to increase the accuracy of SNP genotyping during the quality check, GenCall (GC) and GenTrain (GT) scores were used. GC and GT scores of 0.5 cut-off were applied to determine the valid genotypes for each SNP [31].

The files were merged according to the standard bcftools --merge algorithm with the removal of duplicated SNPs on the same positions. We collected several datasets for complex processing. The first dataset included samples from public databases of wild boars from different parts of the world and those of Russian animals. The second dataset was created to exclude introgression between commercial pig breeds and Russian wild boars. The third dataset included only animals selected on the territory of Russia. The fourth dataset was separated on the basis of ancestry and included four groups: RU_EU and RU_AS animals inhabiting the European and Asian parts of the Russian Federation, respectively, WB_EU and WB_AS animals from different European and Asian regions of the world.

We recommend to select only autosomal chromosomes 1–22, via the PLINK “autosome” option [32]. This option avoids detecting structures that are gender-biased. Filtering genotypes with missing rate > 20% was conducted in PLINK with option “--geno 0.2”. SNPs with high rate of missingness and low minor allele frequency (MAF < 0.01) were removed through the PLINK option “--maf 0.01”. Too rare SNPs (MAF < 0.01) could be found at an individual level but are not commonly present at a population level. Samples with a call rate at least 70% by specifying “--mind 0.3” in PLINK were allowed for analysis. SNP pruning was conducted with filtering out SNPs in linkage disequilibrium (LD) blocks using the PLINK option “--indep-pairwise 50 5 0.2”. We assume low or no correlation structure between SNPs as suggested via r2 < 0.2, with r2 the commonly used measure of LD [33]. LD pruning in this way helps to avoid that strong LD blocks drives the most important calculations, for example—Principal Components or Admixture analysis [34]. Hardy-Weinberg equilibrium test was not performed for comparisons with worldwide population because of Wahlund effect [35]. Upon the completion of filtering, the global and Russian wild boar dataset included 14,491 SNPs (579 samples with 96% genotyping quality), the commercial breed of pig and Russian wild boar—9490 (442 sample with 97% genotyping quality). Homozygous-by-Descent (HBD) Segments of combined groups of European wild boars (WB_EU, *n* = 387) and Asian wild boars (WB_AS, *n* = 34) were compared to Russian European (RU_EU, *n* = 94) and Russian Asian wild boars (RU_AS, *n* = 64).

### 2.3. Genetic Distances and Population Structure

We used PLINK v1.09 to conduct Principal Component Analysis and to visualize the results of comparison inside the selected groups and plotted a scatterplot using the R language. The calculation of Ar (allelic richness); Ho (observed heterozygosity); He (expected heterozygosity); Fis (inbreeding coefficient) were performed using package diversity in R [36]. Average minor allele frequency for each population was calculated with PLINK 1.9. The rate variation among sites was modelled with a gamma distribution (shape parameter = 1) [37]. We applied unsupervised hierarchical clustering of individuals using the maximum likelihood method implemented in ADMIXTURE v.1.3. We used default input parameters for estimation of the admixture components [37]. In addition, ‘--cv’ flag was enabled to perform the cross-validation procedure and to calculate the optimal k value for all populations.

### 2.4. Analysis of Homozygous-by-Descent (HBD) Segments

Realized inbreeding coefficients were calculated using the R package RZooRoH v.0.3.0.74 [38]. This package is based on the hidden Markov model that identifies Homozygous-by-Descent (HBD; evident from ROH) and non-HBD segments. This allows evaluation of the current level of inbreeding better by assuming inbreeding as genome-wide and as local scales, and classifying HBD segments into age-based classes [39]. RZooRoH allows to determine approximate generation classes based on the length of the segments. The different HBD classes are defined by their specific rates Rk. The length of HBD segments from class k is exponentially distributed with the rate Rk and mean 1/Rk. Classes with lower rates correspond to longer HBD segments from more recent common ancestors. Therefore, different HBD classes can be interpreted as HBD segments of different groups of ancestors tracing back to different generations in the past. The rate of the class is approximately equal to the size of the inbreeding loop in generations [39].

## 3. Results

### 3.1. Genetic Diversity and Admixture of the Russian Wild Boar

**Figure 2:**
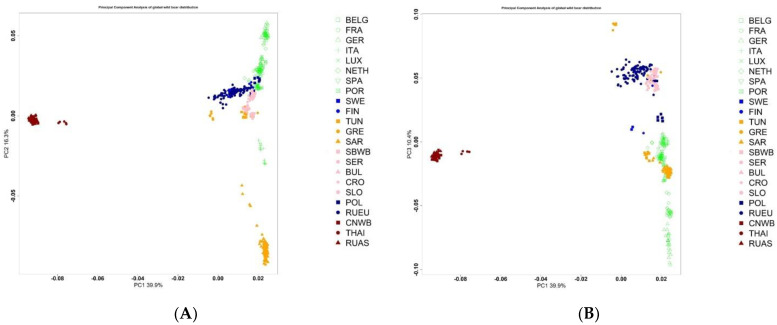

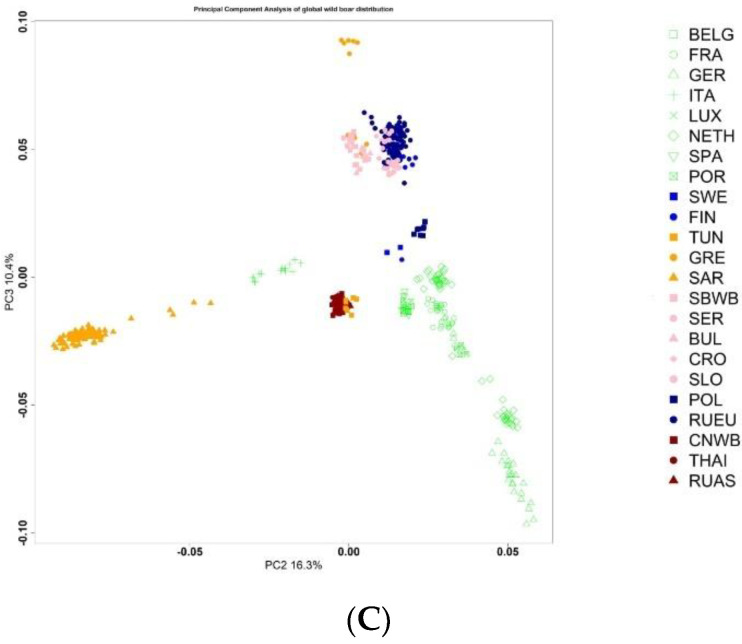
Principal Component Analysis of wild boar from different regions of the world (**A**) PC1 vs. PC2; (**B**) PC1 vs. PC3; (**C**) PC2 vs. PC3.

We assessed the position of the Russian wild boar in the Large Scale Genetic Structure of wild boar from different regions of the world (Figure 2).

As the scatterplot shows, components 1 and 2, as well as 1 and 3, separate the European and Asian boar. The European wild boar from southern regions is distanced from more northern regions, with the exception of a few samples of the SAR population. It is noteworthy that the Russian European wild boar is located in close proximity to the Finnish wild boar and the wild boar of the countries of Eastern Europe. The Russian Asiatic wild boar forms a single group with the Chinese wild boar; wild boars from Thailand form a separate subgroup.

**Figure 3:**
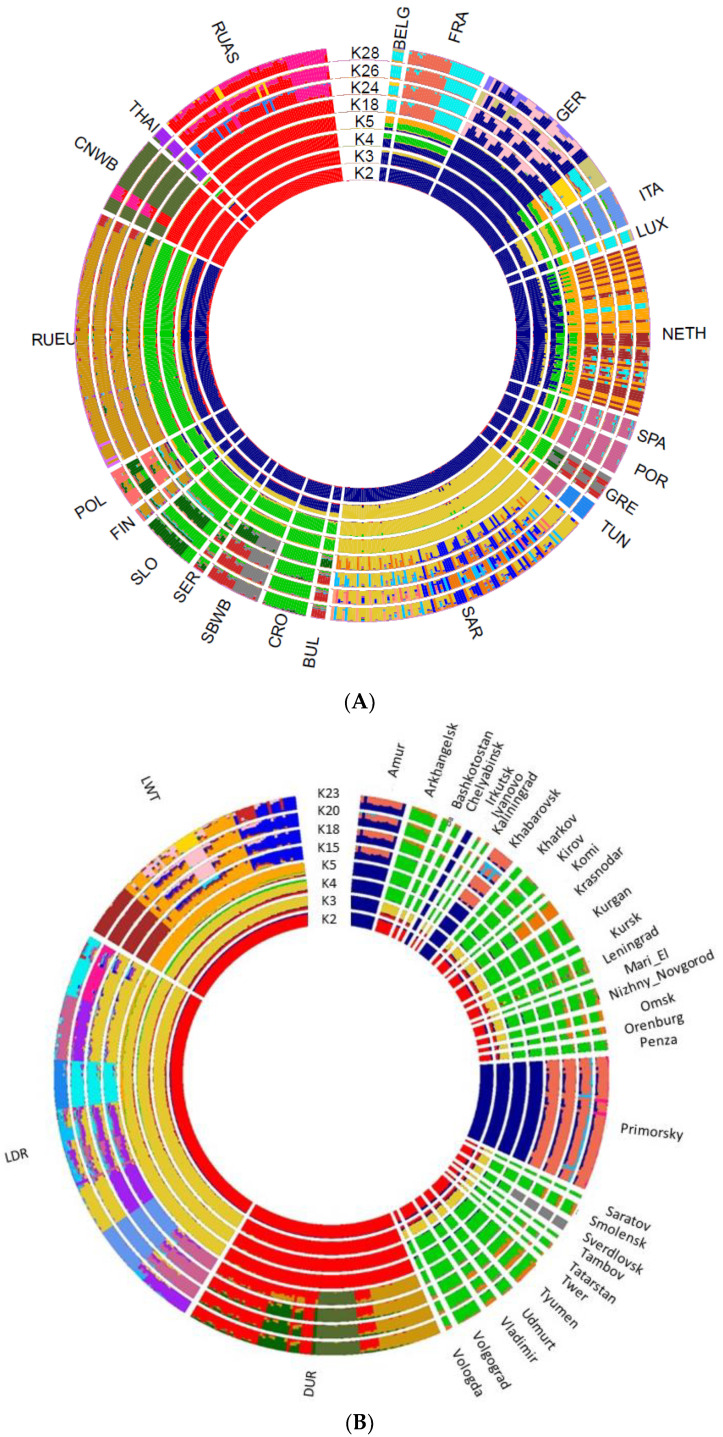
Admixture analysis of wild boar from different regions of the world. Admixture analysis plot in a circular fashion of wild boar from different regions of the world (**A**) and Russian wild boar versus commercial breeds (**B**).

The overall analysis of the population shows a clear division into European and Asian boars at K = 2 (Figure 3A). RUEU boars have traces of the introgression of the Asian boar. At K = 28, both RUAS and RUEU groups of wild boars shows 3 clusters. The minimum Cross-validation error was for K = 18 (Figure S1, Table S2).

We performed an admixture analysis identifying potential hybridization between the Russian wild boar and domestic pigs. Estimation of the parameter Q at K = 5 for which the minimum Cross-validation error was calculated, showed evidence of hybridization between Russian wild boar and domestic pigs for 53 samples of European wild boars. The total share between pig and boar genome was 0.010–0.568 (Figure S2, Table S3). The largest portion of the domestic pig genome was found in a wild boar from the Leningrad region, which was apparently a hybrid of the first generation (0.117 and 0.057 belonged to two clusters of Duroc pigs), and more than 0.100 domestic pig genome was found in four wild boars from Krasnodar.

The observed (Ho) and expected (He) heterozygosity, inbreeding coefficient (F_IS_), and allelic richness (Ar) are shown in Table 1.

The Average Allelic richness varied from 1.40 in the RUAS to 1.70 in the RUEU population. The levels of genetic diversity as indicated by the observed and the expected heterozygosity were similar within the populations of European boars. The lowest values of Ho and He were observed in the RUAS population. The average inbreeding coefficients differed slightly among the populations, being low and positive in the population for RUAS and RUEU. Negative F_IS_ values were observed in BELG, BUL, FRA, LUX, SER, THAI and TUN. The average within-population MAF varied from 0.212 (RUAS) to 0.294 (SER) (Table 1).

### 3.2. Analysis of Homozygous-by-Descent (HBD) Segments

The combined groups of European wild boars (WB_EU), Asian wild boars (WB_AS) were compared to Russian European (RU_EU) and Russian Asian wild boars (RU_AS). The mean level of the inbreeding coefficient of the European wild boar was higher than that of the Asian wild boar, and the combined groups of the European boar had a higher inbreeding coefficient than those of the Russian wild boar (Table 2).

The highest level of total autozygosity was found in the WB_EU group and amounted to 0.168 and the lowest in the RU_AS group was 0.025.

**Figure 4:**
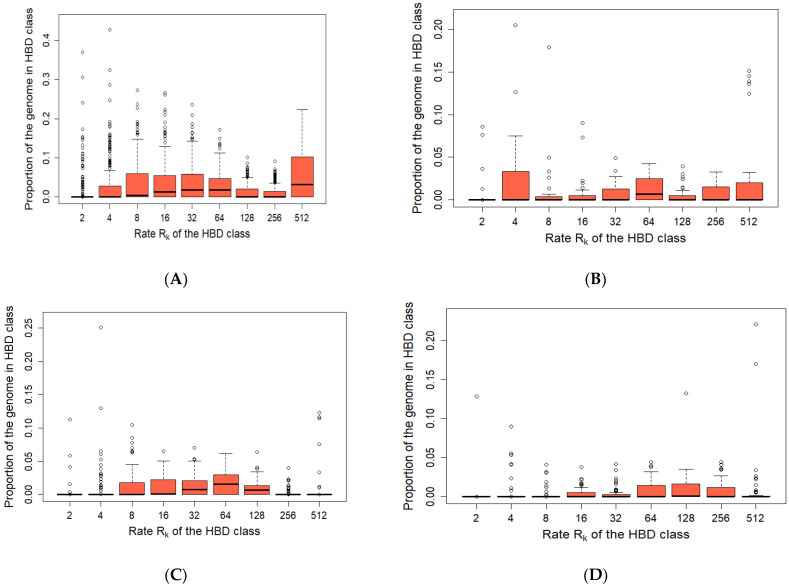
Partitioning of genome-wide autozygosity for wild boar. Boxplot of percentages of individual genomes associated with 9 HBD-classes ((**A**)—WB_EU, (**B**)—WB_AS, (**C**)—RU_EU, (**D**)—RU_AS). The percentages correspond to individual genome-wide probabilities of belonging to each of the HBD-classes.

**Figure 5:**
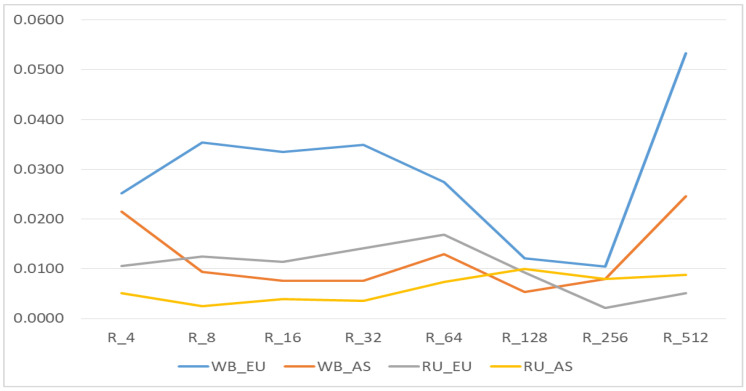
Realized inbreeding coefficients by Homozygous-by-Descent (HBD) classes. European wild boar without Russian European wild boar (WB_EU); Asian wild boar without Russian Asian wild boar (WB_AS); only Russian European wild boar (RU_EU); only Russian Asian wild boar (RU_AS).

Figure 4 and Figure 5 demonstrate the contribution of different HBD segments to total autozygosity.

In the WB_EU group, the short R_512 segments, which show the presence of common ancestors about 250 generations ago, made a greater contribution to autozigosity. In addition, the long and medium R_8—R_64 segments made a rather large contribution. In the WB_AS group, a large share was occupied by long segments R_4, medium segments R_64 and short segments R_256-R_512. In the EU_RU group, there were practically no short segments R_256 and R_512, while larger contribution was made by the middle segments R_8-R_64. In the RU_AS group, although the total share of segments was low the segments of R_64-R_256 classes were prevalent.

The Rk_512_ coefficient in the WB_EU group was 0.053; in the WB_AS group Rk_512_ it was 0.025 (Figure 3 and Figure 4). In the Russian wild boars, it was determined at 0.005–0.009. WB_EU group of wild boars showed higher level of Group inbreeding coefficients by HBD-classes (Figure 4) whereas in other groups the inbreeding was at similar level.

Table 3 presents the results of the number and lengths of segments analysis in groups.

Samples of the WB_EU group are characterized by a larger number of segments compared to other groups (Table 3). In all groups, except for WB_AS, the largest number of detected segments belonged to the Rk_512_ class—22.8–28.5%.

**Figure 6:**
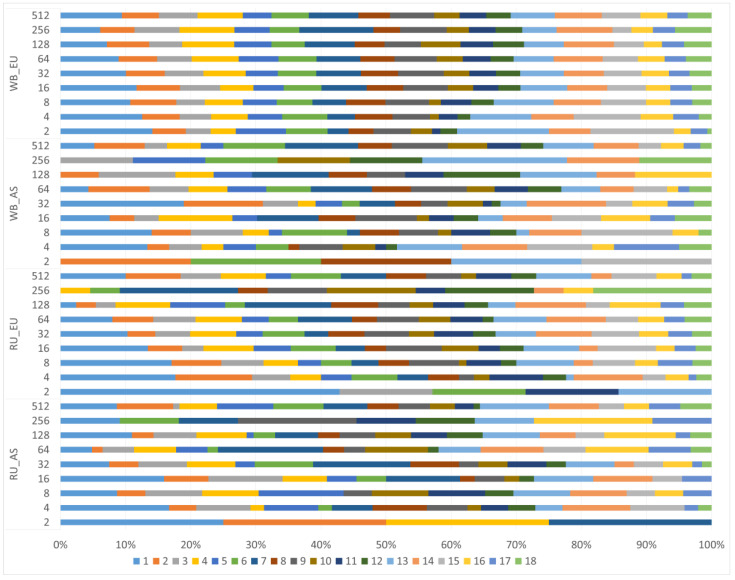
Distribution of segments depending on the class and their location on chromosomes. Chromosomes are shown in different colors.

We examined the distribution of segments depending on the class and their location on the chromosomes (Figure 6).

The profiles of segment distribution of different classes on chromosomes between the groups differed. Long segments were localized mainly on the 1st, 2nd, 3rd, 4th, 6th chromosomes in the Russian wild boars while there were no segments of the Rk2 class on the 1st chromosome in the WB_AS group, but a large proportion of them was recorded on the 2d chromosome, as well as in the RU_AS group. In the WB_EU group, segments of all studied classes were found on all chromosomes.

## 4. Discussion

We investigated the genetic profile of the Russian wild boars, which currently remains poorly studied. There are two subspecies of wild boar that inhabit Russia. The European boars live in the European part of Russia, the Asian boars live in the Primorsky Territory, Amur, Irkutsk Region and Khabarovsk. Our data confirmed (Figure 1 and Figure 2) that European and Asian wild boars differ from each other as indicated by other researchers [20,40,41]. Analysis of genetic profile showed that the European subspecies of the Russian wild boar is characterized by the presence of traces of introgression of the Asian wild boar. This can be explained by the settling of wild boar from the Far East and Primorsky Krai in the territory of the Moscow region (1947–1984), Tver region (1935–1985), Vladimir region (1954–1988), Kaluga region (1964–1981), Yaroslavl region (1961–1986), Nizhny Novgorod region (1963–1969), Volgograd region (1969), Sverdlovsk region (1978–1984) [6]. As shown in Table 1, the parameters of genetic diversity in the European wild boar groups are higher than in the Asian wild boar groups. Minor Allele Frequency (MAF), is an indicator of the abundance of rare alleles through the genome. In our study it ranged from 0.212 to 0.294 in our study, for Spanish was wild boars—0.192 [42], for different populations of European wild boar was in the range of 0.136–0.174 [43], the average MAF within local breeds ranged from 0.133 to 0.294 [42,43], for domestic pig of Landrace and Large White was breeds higher than 0.250 [44]. Xiao et al. showed that a low MAF can indicate a vulnerable position of population while high MAF in a small population can be a sign of high tolerance for inbred depression [45]. We found negative inbreeding coefficient (Fis) values for wild boar groups from Belgium, Bulgaria, France, Luxembourg, Serbia, Thailand and Tunisia, apparently this is due to the small size of the groups. Sánchez-Montes et al. pointed out the influence of population size and the presence of close relatives on the parameters of genetic diversity [46]. Analysis of the genetic diversity of the Russian European wild boar as compared with world-wide population showed the highest rate of allelic diversity, heterozygosity, while the genetic diversity of the Russian Asian wild boar is reduced. Slightly higher genetic diversity of the Russian European boar were indicated by Ho and He data that were for RUAS 0.185 and 0.190, for RUEU 0.328 and 0.342, for the wild boar from Iran 0.284 and 0.249; for the wild boar from Eastern Europe 0.325 and 0.312, for the wild boar from Western Europe 0.301 and 0.296 [47]. The study of the genetic diversity of the Asian wild boar using microsatellites showed that the allelic diversity and the level of heterozygosity of the Russian Asian wild boar were lower than those of the Chinese wild boar [16]. Analysis of a polymorphism of mtDNA showed that the level of genetic diversity of wild boars is higher in East Asia than in Europe [48].

Iacolina et al. [43] did not find hybrids in the Central-North-Eastern Europe wild boar population and also among four sample of Russian wild boars, but many researchers indicate traces of introgression of wild boar and domestic pigs [43,49,50,51]. We analyzed the Russian wild boars and commercial breeds of pigs and did not find any traces of hybridization with Asian wild boars, but we found strong traces of such hybridization for one sample from Leningrad Region and less strong for 4 boars from Krasnodar. Goedbloed et al. [51] found for geographically widespread presence of domestic pig SNPs in 10% of analyzed wild boars of Northwest Europe. The lack of hybrids among the Asian wild boars can be explained by the more severe environmental conditions that are not favorable for pig breeding. On the territory of the European part of Russia, there are more favorable conditions for non-industrial pig breeding; accordingly, there is a high probability of hybridization of wild boar with domestic pigs.

Since the wild boar in the European part of Russia was practically exterminated and restored and expanded its habitat since the second half of the 20th century, it is important to understand the level of autozygosity in the modern wild boar population [6]. We examined HBD segments to assess the autozygosity of the Russian European and Russian Asian wild boar. The level of total autozygosity in Russian populations of wild boar was lower than in other groups. When compared with domestic pigs, the level of autozygosity of wild boar was lower. The studies of Landrace and Large White pig showed that total autozygosity (inbreeding coefficient) was approximately 0.23 (0.15–0.34) [52], for different populations of wild boar was in the range of 0.025–0.168. Short segments are practically absent in Russian wild boar. This can be explained by the relatively recent recovery of the modern wild boar population in Russia. The total number of segments in domestic pigs is also significantly higher, approximately 30,000–50,000, although more than 28,000 of segments have been identified in the European wild boar group (73.56 segments per an individual), but no more than 2000 segments have been identified in other wild boar groups (5.33–55.71 segments per an individual). Our studies have shown that HBD segments are evenly distributed across chromosomes in WB_EU, as previously reported by Bosse et al. [53], which also indicates that Chinese boars represent the most variable cluster due to their high nucleotide diversity and low amount of ROH (*p* < 0.001). In our case, the Asian boars had significantly fewer HBD segments than the European boars.

Our study investigated a genetic profile of the Russian wild boar population, determined the level of autozygosity, found the introgression of the domestic pig genome into European Russian boar and showed that the Asian Russian boar population is in a vulnerable position due to reduced genetic diversity.

## 5. Conclusions

Our study provides a comparative analysis of the genetic diversity of wild boars of various origins. Using various methods of processing genome-wide genotyping data, it was demonstrated that wild boars from different regions of the world differ in their genetic structure and the level of autozygosity. We found traces of domestic pig introgression in the Russian European wild boar. Our results can be useful for characterizing different populations of wild boar and domestic pigs. They provide important information for the conservation and management of biodiversity and expand understanding of the genetic state of the Russian wild boar population.

## Figures and Tables

**Table 1:** Population genetics analyses of Wild boar from different regions.

Pop	N	MAF	Nsnp	Ar	Ho	He	Fis
BELG	4	0.295 ± 0.0013	10,423	1.65 ± 0.005	0.311 ± 0.0023	0.276 ± 0.0016	−0.128 ± 0.0044
BUL	5	0.285 ± 0.0012	11,211	1.67 ± 0.004	0.324 ± 0.0021	0.289 ± 0.0015	−0.120 ± 0.0037
CNWB	29	0.243 ± 0.0014	9780	1.47 ± 0.004	0.208 ± 0.0016	0.228 ± 0.0017	0.055 ± 0.0021
CRO	16	0.270 ± 0.0013	12,183	1.64 ± 0.004	0.297 ± 0.0017	0.299 ± 0.0015	0.002 ± 0.0022
FIN	5	0.290 ± 0.0012	11,546	1.69 ± 0.004	0.294 ± 0.0020	0.301 ± 0.0015	0.010 ± 0.0041
FRA	28	0.270 ± 0.0012	12,794	1.66 ± 0.004	0.313 ± 0.0016	0.313 ± 0.0015	−0.001 ± 0.0020
GER	57	0.251 ± 0.0013	12,398	1.60 ± 0.004	0.278 ± 0.0016	0.285 ± 0.0015	0.031 ± 0.0020
GRE	9	0.272 ± 0.0012	12,096	1.66 ± 0.004	0.275 ± 0.0017	0.300 ± 0.0015	0.065 ± 0.0032
ITA	15	0.264 ± 0.0013	11,892	1.62 ± 0.004	0.266 ± 0.0016	0.287 ± 0.0015	0.060 ± 0.0025
LUX	4	0.295 ± 0.0013	10,423	1.65 ± 0.005	0.311 ± 0.0023	0.276 ± 0.0016	−0.128 ± 0.0044
NETH	62	0.264 ± 0.0013	13,246	1.66 ± 0.004	0.260 ± 0.0013	0.316 ± 0.0014	0.164 ± 0.0018
POL	14	0.283 ± 0.0012	12,347	1.67 ± 0.004	0.308 ± 0.0018	0.314 ± 0.0015	0.015 ± 0.0031
POR	11	0.270 ± 0.0013	11,672	1.62 ± 0.004	0.275 ± 0.0017	0.287 ± 0.0015	0.028 ± 0.0029
RUAS	64	0.212 ± 0.0016	9600	1.40 ± 0.004	0.185 ± 0.0016	0.190 ± 0.0016	0.027 ± 0.0017
RUEU	94	0.280 ± 0.0013	13,783	1.70 ± 0.004	0.328 ± 0.0014	0.342 ± 0.0014	0.043 ± 0.0011
SAR	99	0.236 ± 0.0013	13,439	1.61 ± 0.004	0.245 ± 0.0013	0.291 ± 0.0015	0.148 ± 0.0013
SBWB	20	0.272 ± 0.0012	12,819	1.68 ± 0.004	0.289 ± 0.0015	0.323 ± 0.0015	0.080 ± 0.0022
SER	4	0.296 ± 0.0012	10,609	1.66 ± 0.005	0.322 ± 0.0024	0.281 ± 0.0016	−0.142 ± 0.0045
SLO	20	0.278 ± 0.0012	12,458	1.66 ± 0.004	0.307 ± 0.0016	0.312 ± 0.0015	0.013 ± 0.0021
SPA	7	0.281 ± 0.0012	11,786	1.67 ± 0.004	0.286 ± 0.0018	0.300 ± 0.0015	0.032 ± 0.0035
THAI	5	0.282 ± 0.0016	7586	1.44 ± 0.005	0.231 ± 0.0024	0.200 ± 0.0018	−0.143 ± 0.0055
TUN	7	0.264 ± 0.0014	9954	1.57 ± 0.004	0.262 ± 0.0020	0.275 ± 0.0019	−0.053 ± 0.0035

Legend: populations of wild boar (Pop); Samples sizes (N); mean minor allele frequencies (MAF); the number of SNP (Nsnp); allelic richness (Ar); observed heterozygosity (Ho); expected heterozygosity (He); inbreeding coefficient of an individual (I) relative to the subpopulation (S) (F_IS_), wild boars from Tunisia (TUN), Belgium (BELG), Luxemburg (LUX), Portugal (POR), Spain (SPA), France (FRA), Germany (GER), Netherlands (NETH), Greece (GRE), Italy (ITA), Sardinia Islands (SAR), South Balkan wild boars (SBWB), Sweden (SWE), Finland (FIN), Serbia (SER), Bulgaria (BUL), Croatia (CRO), Slovenia (SLO,), Poland (POL), Thailand (THAI), Chinese wild boar (CNWB).

**Table 2:** Summary statistics of the inbreeding coefficient.

	WB_EU	WB_AS	RU_EU	RU_AS
Min	0.000	0.008	0.009	0.000
1st Qu.	0.086	0.024	0.038	0.008
Median	0.151	0.040	0.060	0.018
Mean	0.168	0.065	0.068	0.025
3rd Qu.	0.230	0.063	0.078	0.034
Max.	0.554	0.354	0.354	0.143

**Table 3:** Number and length (Mb) of the Homozygous-by-Descent (HBD) segments.

Rk	WB_EU		WB_AS		RU_EU		RU_AS	
	N_t (N_i)	Length	N_t (N_i)	Length	N_t (N_i)	Length	N_t (N_i)	Length
2	156 (0.40)	80.76 ± 3.889	6 (0.18)	98.78 ± 24.633	5 (0.05)	114.55 ± 13.938	4 (0.06)	91.81 ± 42.738
4	661 (1.71)	42.44 ± 1.082	85 (2.50)	33.51 ± 2.719	60 (0.64)	36.93 ± 3.585	48 (0.76)	24.03 ± 2.398
8	1876 (4.85)	20.47 ± 0.323	170 (5.00)	18.14 ± 0.902	50 (0.53)	16.44 ± 1.710	23 (0.37)	14.72± 1.733
16	3448 (8.91)	10.12 ± 0.113	246 (7.24)	10.82 ± 0.360	53 (0.56)	9.81 ± 0.749	46 (0.73)	12.40± 0.744
32	5967 (15.42)	5.20 ± 0.400	467 (13.74)	5.32 ± 0.129	74 (0.79)	6.53 ± 0.357	67 (1.06)	7.22 ± 0.507
64	5508 (14.23)	2.97 ± 0.200	603 (17.74)	3.05 ± 0.060	117 (1.24)	3.287 ± 0.132	62 (0.98)	3.99 ± 0.200
128	1877 (4.85)	1.65 ± 0.016	166 (4.88)	1.66 ± 0.059	17 (0.18)	2.06 ± 0.273	91 (1.44)	2.57 ± 0.122
256	852 (2.20)	0.85 ± 0.014	21 (0.62)	0.72 ± 0.092	9 (0.10)	0.83 ± 0.095	11 (0.17)	0.71 ± 0.125
512	8122 (20.99)	0.41 ± 0.004	130 (3.82)	0.32 ± 0.021	116 (1.23)	0.30 ± 0.030	104 (7.65)	0.29 ± 0.030
All	28,467 (73.56)	5.92 ± 0.710	1894 (55.71)	7.31 ± 0.271	501 (5.33)	10.13 ± 0.843	456 (7.23)	7.53 ± 0.665

Legend: Rate of class (Rk); Total number of segments in population (N_t); number of segments pro individual (N_i); mean length of segments (Lenght); European wild boar without Russian European wild boar (WB_EU); Asian wild boar without Russian Asian wild boar (WB_AS); only Russian European wild boar (RU_EU); only Russian Asian wild boar (RU_AS).

## Data Availability

The raw data supporting the conclusions of this article will be made available by the authors upon reasonable request.

## Supplementary Materials

The following supporting information can be downloaded at: https://www.mdpi.com/article/10.3390/biology11020203/s1. Figure S1: cv_all_wild_boars CV, Figure S2: cv_dom_CV, Table S1: Samples and genotyping panels, Table S2: Parameter Q K = 15 domestic and russian wild boar, Table S3: Parameter Q K = 18 all wild boars.
