# Supplementary figures and images for: Genetic Diversity, Admixture and Analysis of Homozygous-by-Descent (HBD) Segments of Russian Wild Boar
Source: Biology (Basel). 2022 Jan 27;11(2):203. doi: 10.3390/biology11020203 (PMC8869248; doi:10.3390/biology11020203)

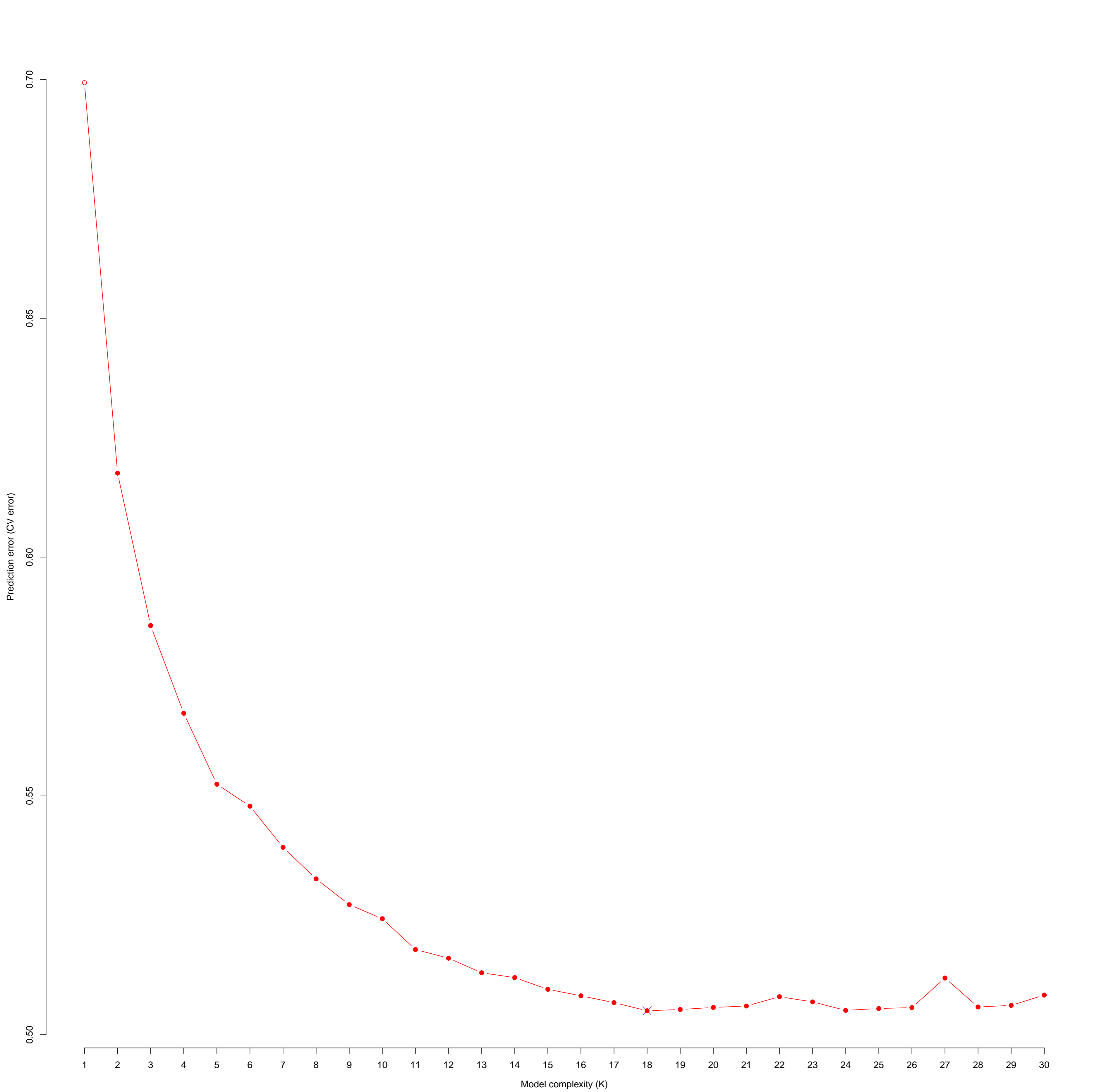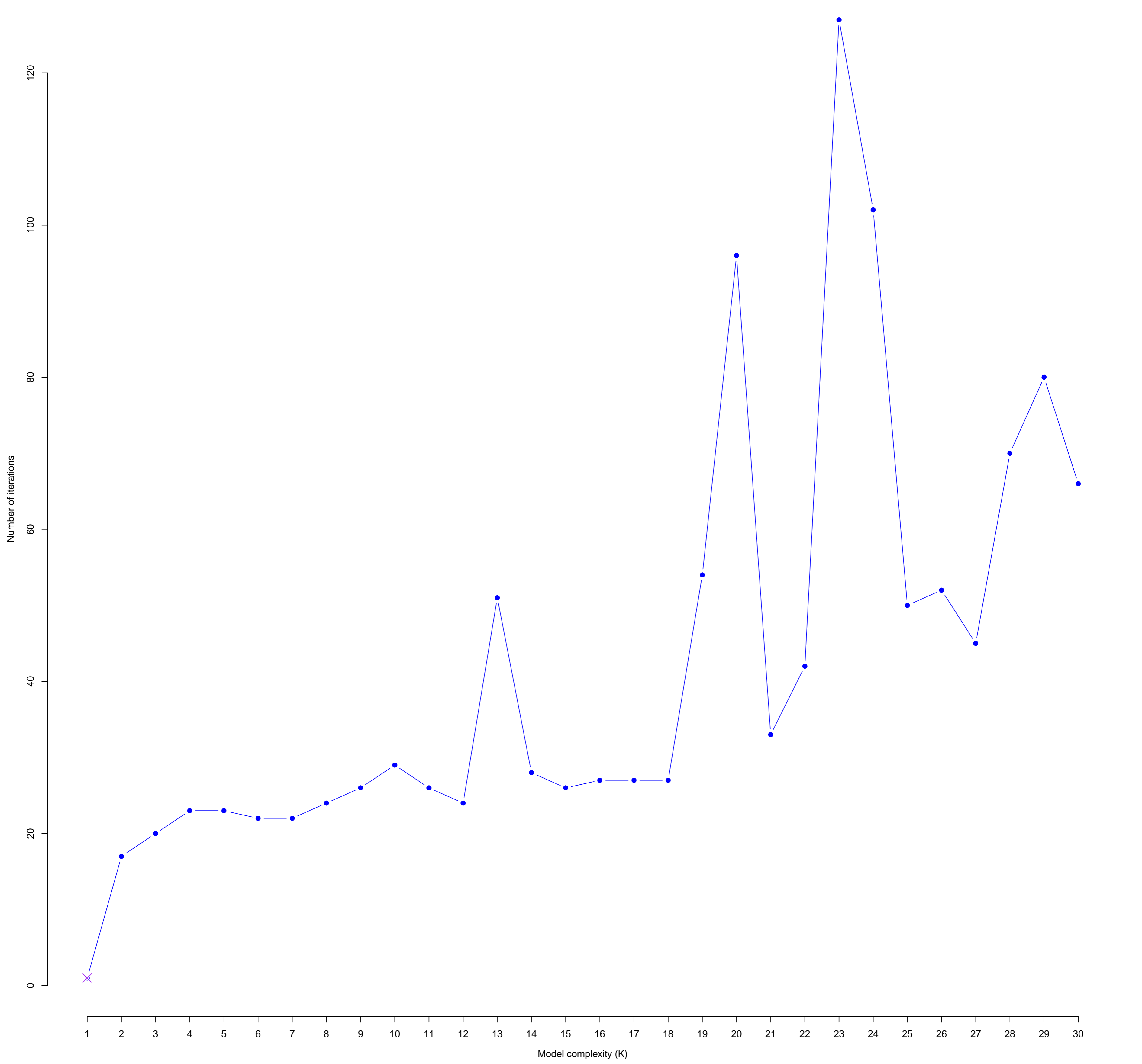

Supplement: Supplementary file 1 [file biology-11-00203-s001.zip › Figure S1. cv_all_wild_boars CV.pdf]

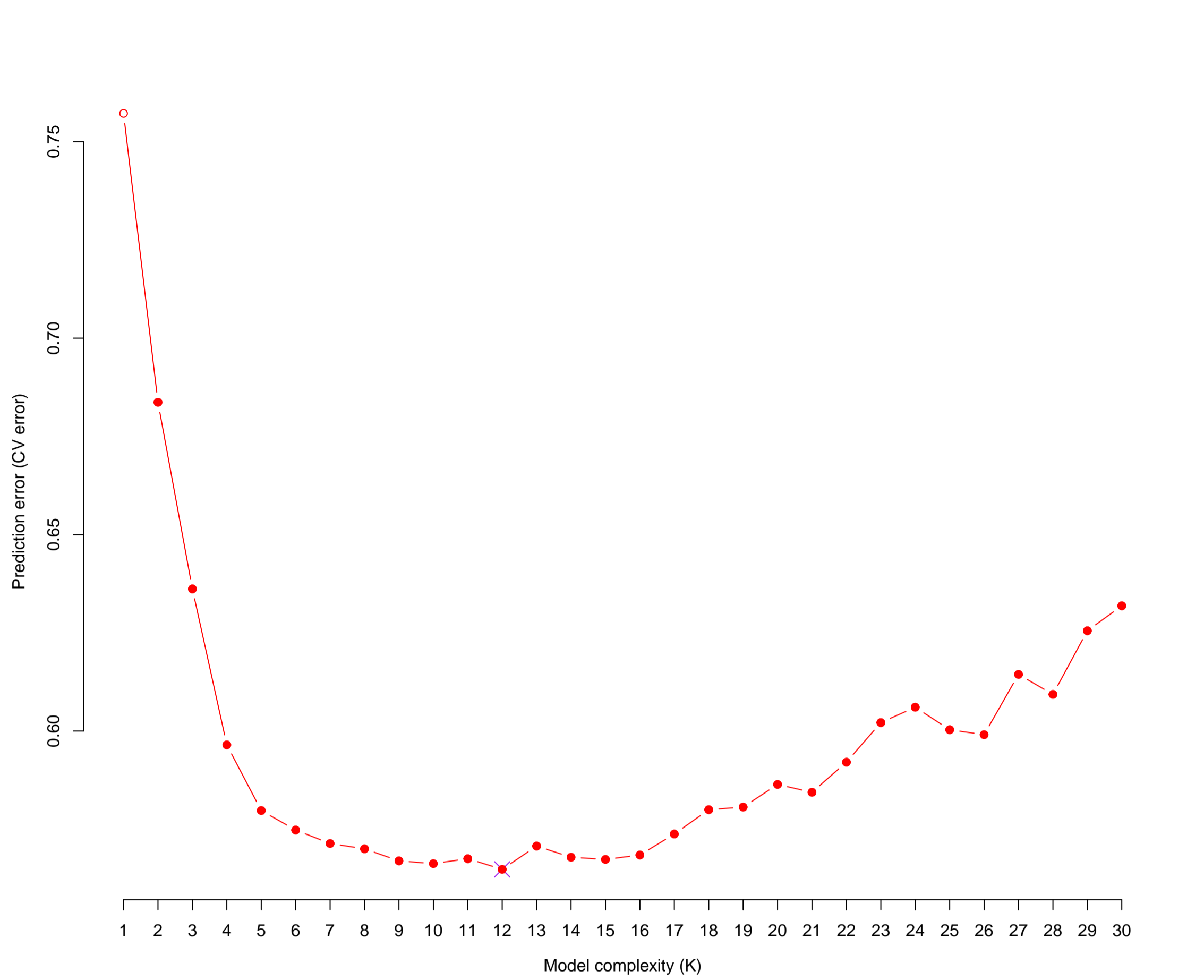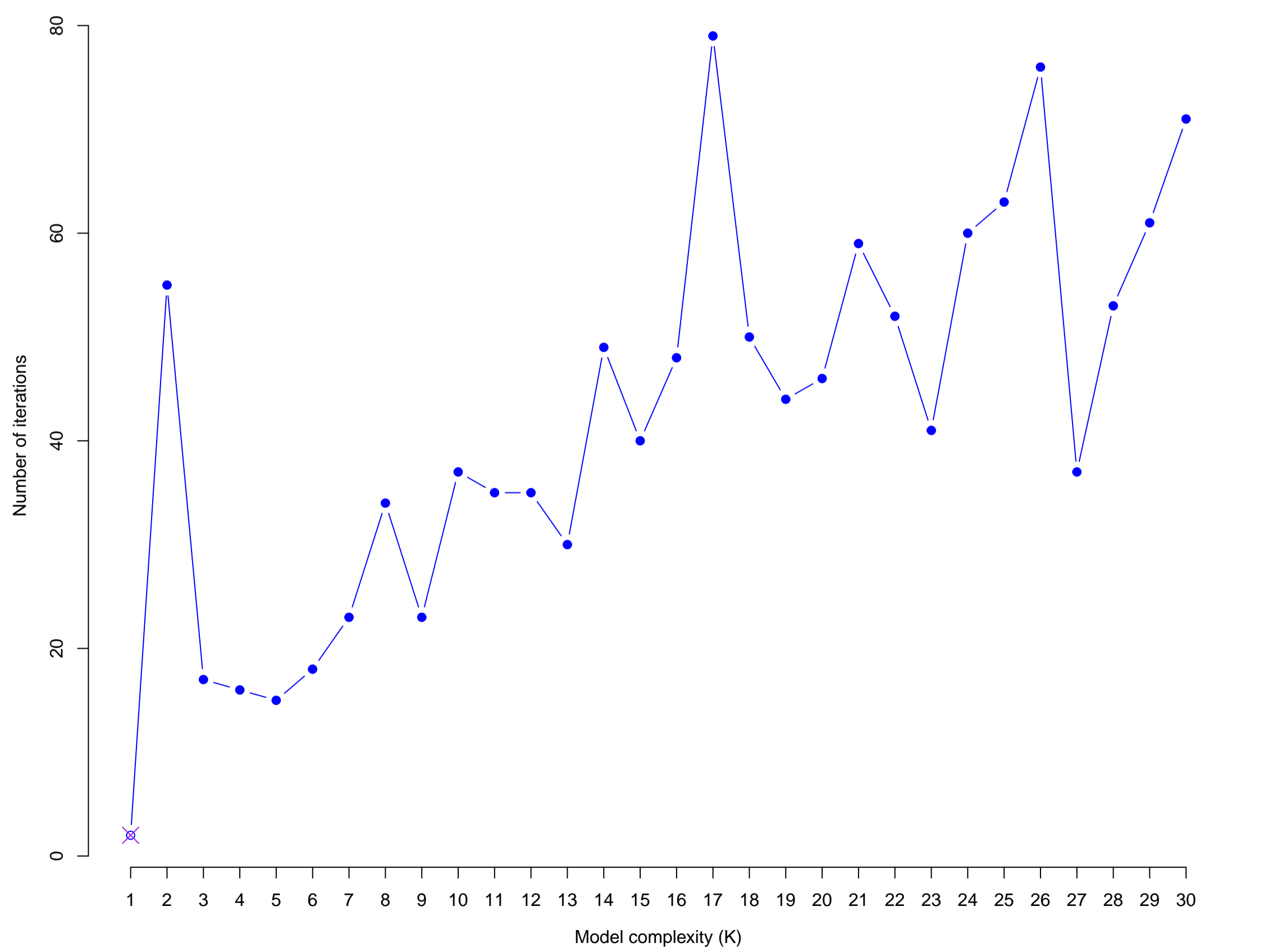

Supplement: Supplementary file 1 [file biology-11-00203-s001.zip › Figure S2. cv_dom_CV.pdf]
